# Secondary Prevention by Enhancing Adherence in Diabetic Patients

**Published:** 2010

**Authors:** Habibeh Ahmadipour, Ziba Farajzadegan, Ali Kachoei, Azar Pirdehghan

**Affiliations:** 1 Assistant Professor of Community Medicine, Kerman University of Medical Sciences, Kerman, Iran; 2 Associate Professor of Community Medicine, Isfahan University of Medical Sciences, Isfahan, Iran; 3 Associate Professor of Endocrinology, Isfahan University of Medical Sciences, Isfahan, Iran; 4 Resident of Community Medicine, Isfahan University of Medical Sciences, Isfahan, Iran

**Keywords:** Diabetes, Adherence, Prevention, Complication

## Abstract

**Objectives:**

Adherence to prescribed medications is a key dimension of healthcare quality. Poor medication adherence might be a significant barrier to achievement of positive clinical outcomes. This study aimed to compare the adherence to oral hypoglycemic agents in patients with type 2 diabetes by using two methods of completing diary checklist and collecting drug shells.

**Methods:**

This randomized clinical trial was conducted in Isfahan Diabetes Institute. A number of 100 type 2-diabetic patients were selected through systematic sampling method and then were randomly allocated to two groups of equal number. Each group was invited to attend our 12-week educational program. We asked one group to complete diary checklist about how they took their drugs during the study period. The other group was asked to collect the shells of oral hypoglycemic agents after taking in a pocket. Medication adherence ratio was calculated for both groups.

**Results:**

Overall, 87 patients completed the study, of which 30 cases (34.5%) were in the check list group and 57 (65.5%) in the reference group. In the check list group, the adherence ratio was good in 96.7% and moderate in 3.3%, with no case of poor adherence. In the reference group, the corresponding figures were 55.2%, %6.9 and 37.9%, respectively (p<0.05 between groups).

**Conclusions:**

Although the adherence ratio was greater than 80% in both groups, it was significantly higher in the check list group. Therefore, we suggest that by increasing adherence to prescribed medications, diary checklist can be an effective method of secondary prevention of chronic diseases, as diabetes mellitus.

## INTRODUCTION

Adherence to prescribed medications is a key dimension of healthcare quality.^1^ Adherence to (or compliance with) a medication regimen is generally defined as the extent to which patients take medications as prescribed by their health care providers. The word “adherence” is preferred by many health care providers, because “compliance” suggests that the patient is passively following the doctor’s orders and that the treatment plan is not based on a therapeutic alliance or contract established between the patient and the physician. Both terms are imperfect and uninformative descriptions of medicationtaking behavior. Unfortunately, applying these terms to patients who do not consume every pill at the desired time can stigmatize these patients in their future relationships with health care providers. The language used to describe how patients take their medications needs to be reassessed, but these terms are still commonly used.^2^ Non-adherence is associated with poor health outcomes^1^ and with substantial economic cost^2^,^3^ and threatens the gains in quality that have been made by appropriate pharmacotherapy over the past several decades.^4^ Efforts to accurately measure and improve adherence have received increasing attention from patients, physicians, payers, and other healthcare stakeholders. Moreover, the National Committee for Quality Assurance has recently included adherence among the measures by which it evaluates the quality of care provided by healthcare plans.^5^ Adherence to medication regimens has been monitored since the time of Hippocrates, when the effects of various options were recorded with notations of whether the patient had taken them or not. Even today, patients’ self-reports can simply and effectively measure adherence.^6^,^7^

The methods available for measuring adherence can be broken down into direct and indirect methods of measurement. Each method has advantages and disadvantages, and no method is considered the gold standard.^8^,^9^ Directly observed therapy, measurement of concentrations of a drug or its metabolite in blood or urine, and detection or measurement in blood of a biologic marker added to the drug formulation are examples of direct methods of measures of adherence. Direct approaches are expensive, burdensome to the health care provider, and susceptible to distortion by the patient.

Indirect methods of measurement of adherence include asking the patient about how easy it is for him or her to take prescribed medication, assessing clinical response, performing pill counts, ascertaining rates of refilling prescriptions, collecting patient questionnaires, using electronic medication monitors, measuring physiologic markers, asking the patient to keep a medication diary, and assessing children’s adherence by asking the help of a caregiver, school nurse, or teacher. Questioning the patient (or using a questionnaire), patient diaries, and assessment of clinical response are all methods that are relatively easy to use, but questioning the patient can be susceptible to misrepresentation and tends to result in the health care provider’s overestimating the patient’s adherence.^10^–^12^

Adherence rates are typically higher among patients with acute conditions, as compared with those with chronic conditions; persistence among patients with chronic conditions is disappointingly low, dropping most dramatically after the first six months of therapy.^13^–^15^

Non-adherence to diabetes treatment leads to poor glucose control and increases the risk of disease complications. The prevalence and factors associated with non-adherence in resource limited settings should be determined so as to lower the impact of a disease that is on the increase, on the health systems which are already overburdened with communicable diseases.^16^ There are many factors that contribute to successful blood glucose control, including appropriate diet, exercise goals, and patient motivation. Oral medications also play an important role in the management of type 2 diabetes. With evidence linking such pharmacological modalities to better outcomes, awareness of the critical role of adherence to pharmacologic therapy has been heightened. A recent meta-analysis showed that the average adherence to therapy in patients with diabetes is 67.5%, which is lower than that seen with various other conditions such as human immunodeficiency virus disease, osteoarthritis, gastrointestinal disorders, and cancer.^17^ Poor medication adherence would seem to be a significant barrier to attainment of positive clinical outcomes such as a decrease in both micro and macrovascular disease.^18^

A study was noted that only 25% of the study group was adhering to the treatment regularly. Dietary prescriptions were followed regularly only by thirty seven percent. Home glucose monitoring was being done only by twenty three percent. Non adherence was not related either to the age or duration of diabetes. Non adherence was more in the lower socio-economic group and was inversely related to the educational status.^19^

Depressed patients have lower adherence rates than non depressed patients (85 vs. 93%, respectively).^20^ Once-daily regimens have higher adherence than twice-daily regimens (61 vs. 52%, respectively).^21^ Monotherapy regimens have higher adherence than polytherapy regimens (49 vs. 36%, respectively)^20^ or a higher proportion of patients achieving high adherence ratios (35 vs. 27% at 90% or higher adherence ratios)^22^ Patients converting from monotherapy or polytherapy to a single combination tablet improved their adherence rates by 23 and 16%, respectively.^23^

Even though adherence aids are in common use among adults with diabetes, there is little evidence that they are effective. In a study, a few associations between adherence aids with better control were observed, but they may be artifacts of multiple comparisons or unmeasured confounders.^24^

The purpose of this study was to compare the adherence to oral hypoglycemic agents in patients with type 2 diabetes by using two methods of completing diary checklist and collecting drug shells.

## METHODS

This randomized clinical trial was carried out in Isfahan Diabetes Institute. Participants were selected from the patients list of the Institute through systematic sampling method and then they were randomly allocated to 2 groups.

Inclusion criteria were patients with type 2 diabetes mellitus taking oral hypoglycemic agents (OHAs), at least 1 year passed from the onset of treatment and giving informed consent. Exclusion criteria were any change in the current treatment program, patient request to not continue the study and incomplete collecting the drug shells or checklists. Each group members were invited to attend in educational program held by the aforementioned institute about importance of adherence to drugs and consequences of poor glycemic control.

To reach the purpose of the trial, one group was asked to complete diary checklist about how they took their drugs during the study period. The other group was asked to collect the shells of oral hypoglycemic agents after taking in a pocket. Hemoglobin (Hb)A1c was measured in both groups by using chromatographic method at the onset of the study as a baseline. The study duration was 12 weeks. Then the checklists and pockets collected and Hb A1c was measured another time as an actual outcome. Medication adherence ratio was calculated for both groups. This ratio was estimated by dividing sum of the drugs patients should take according to their treatment program per sum of the drugs they reported to take.

### Statistical analysis

We used independent t test and X^2^ for analysis, then adjustment for sex, age, education level, hypertension, hyperlipidemia and baseline hemoglobinA1c was done by Multivariate analysis of covariance (MANCOVA). Cut of point of adherence ratio in our study was 80%.The ratios lower than 80% determined as poor, 80-90 % as medium and >90% as adequate adherence. Data was analyzed by using SPSS statistical package version 15.0 for windows (SPSS Inc., Chicago, USA).

## RESULTS

**Figure 1:**
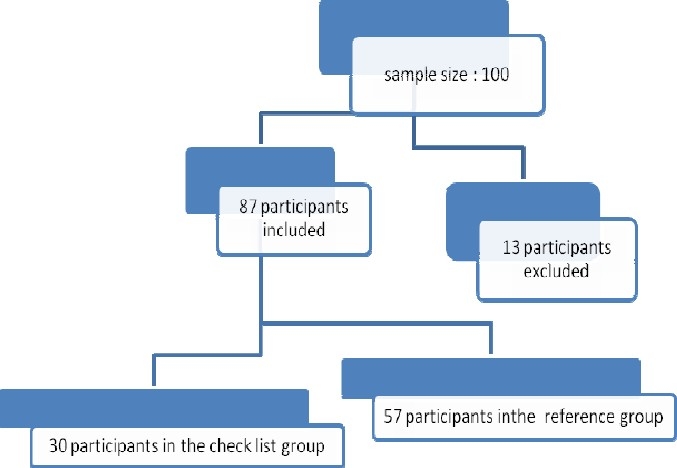
Retention vs. attrition in the study participants

As presented in Figure 1, 87 patients with type 2 diabetes were studied, of which 30 cases (34.5%) were in the check list group and 57 (65.5%) in the reference group.

**Figure 2:**
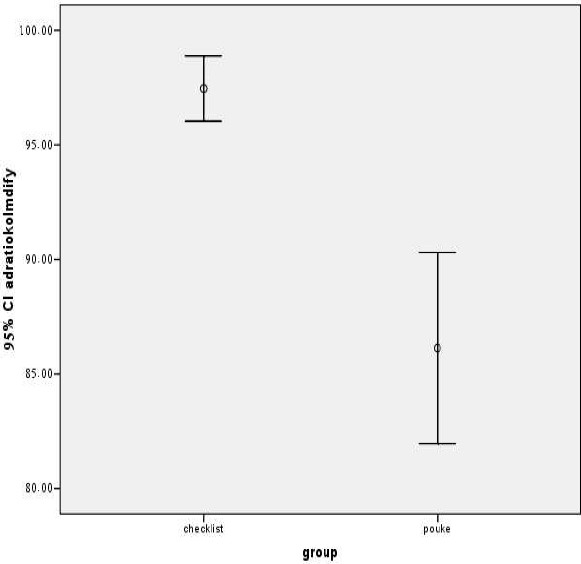
Comparison of adherence between two groups at the end of the trial

Demographic characteristics of patients in group care are in Table 1. In the check list group, adherence was good in 96.7% and mode ratio in 3.3% of patients, with no case of poor adherence. The corresponding figures in the reference group were 55.2%, 6.9% and 37.9%, respectively.

**Table 1:** Demographic characteristics of diabetic patients in two groups

	Check list group	Reference group		pvalue
Age				
	48.1±7.81 years	52.3±8.52years	T=-2.09	0.04
Sex			X=0.89	0.3
Female	82.1%(n=23)	89.5%(n=51)		
Male	17.9%(n= 5)	10.5%(n= 6)		
Level of education			X=9.38	0.009
Lower than high- school diploma	66.7%(n=16)	92.7%(n=51)		
High school diploma	29.2%(n=7)	7.3%(n=4)		
University Graduate	4.2%(n= 1)	0%(n= 0)		
HbA1c	8.1±1.55%	7.8±1.51%	T=0.62	0.5

There was a significant relationship between adherence and the type of group (K2 = 16, p= 0.001). In other words, the mean of adherence was 97.4± 3.81 and 86.1±15.7 in the check list and the reference group, respectively (t = 3.8, p< 0.0001).

Among females, adherence to medication was 97.1±4.14 and 86.5±15. %1 in the check list and reference groups, respectively (t = 3.1, p = 0.002). The corresponding figures in males were 98.6 ±2.59 and 82.6 ±21.1%, respectively. (t = 1.6, p = 0.13).

Considering the education level, in those patients with education level of equal or lower than high-school, adherence was significantly higher in the check list than in the reference group (Table 1).

Comparison of each group separately, showed that the mean of adherence was not significantly different according to education level.

There was a non-significant inverse relationship between adherence and age (Pearson correlation = - 0.03, p = 0.76).

The difference between the mean of hemo-globin A1C level before and after intervention was not significant between groups. This difference was not significant in terms of gender and age group.

As presented in Figure 1, there was significant difference in the mean of adherence between two groups. (p = 0.02).

## DISCUSSION

In spite of the importance of adherence to drug treatment, it is estimated that only about half the people who leave a doctor’s office with a prescription, would take the drug as directed. Among the many reasons people give for not adhering to drug treatment, forgetfulness is the most common. The purpose of our study was to compare the adherence to oral hypoglycemic agents in patients with type 2 diabetes mellitus by 2 methods of completing diary checklist and collecting drug shells, as reference. Our hypothesis was that diary checklist could increase adherence to drug by reducing forgetfulness. In the current trial, we found that adherence ratio in both groups were greater than 80%, in other words both groups had medium and high adherence, which increased after using the checklist method. There is no consensual standard for what constitutes adequate adherence. Some trials consider rates of greater than 80 percent to be acceptable, whereas others consider rates of greater than 95 percent to be mandatory for adequate adherence, particularly among patients with serious conditions such as infection with the human immunodeficiency virus (HIV). Although data on adherence are often reported as dichotomous variables (adherence vs. non adherence), adherence can vary along a continuum from 0 to more than 100 percent, since patients sometimes take more than the prescribed amount of medication.^11^,^25^,^26^

The average ratios of adherence in clinical trials can be remarkably high, owing to the attention study patients receive and to selection of the patients, yet even clinical trials report average adherence ratios of only 43 to 78 percent among patients receiving treatment for chronic conditions.^26^,^27^,^28^

A systematic review of adherence to medications for diabetes showed that the average adherence to oral antihyperglycemic medications ranged from 36 to 93% for patients who remained on treatment for 6 to 24 months.^29^

We found that in both groups, the adherence ratio of females was equal or higher than males, although this difference did not reach significance level, but this finding is not compatible with some previous studies. In the study of Hertz et al., female gender increased the risk for early non-persistence and for drug discontinuation over time.^30^ Bhattacharya et al. found that adherence was better in males, in married and educated individuals.^31^ It can be assumed that the higher adherence of females in our study might be because of the greater numbers of female participants with more attendance in educational programs held by the Institute. In both genders, the adherence ratio was higher in the check list than in the reference group.

Although not significant, but in both groups we found an inverse association between education level and adherence to drugs. This finding is similar to the study of Herz et al.^30^ We found a not significant inverse relation between age and adherence to drugs. Hertz et al. found that younger age, i.e., 18-24 years, increased the risk for early non persistence and for drug discontinuation over time.^30^ Bhattacharya and colleagues showed that compliance was better in those patients aged above 60 years. ^31^ In the study of Shobhana and colleagues, non adherence was not related to the age.^19^ After adjustment for confounding variables, adherence ratio was greater in checklist group. Probably, this method is more effective for increasing adherence to prescribed drugs in patients.

## CONCLUSIONS

We found that diary checklist can be an effective method for increasing adherence to prescribed drugs of secondary prevention of chronic diseases, as diabetes mellitus.
